# Epidemiological Trends and Factors Associated With Mortality Rate in Psychoactive Substance Use-Related Mental and Behavioral Disorders: A CDC-WONDER Database Analysis

**DOI:** 10.7759/cureus.49647

**Published:** 2023-11-29

**Authors:** Okelue E Okobi, Ngozi T Akueme, Amogechukwu O Ugwu, Imoh L Ebong, Nnena Osagwu, Lilian Opiegbe, Ibrahim L Folorunsho, Linda A Mbah, Gabriel I Ude, Ambar Khan, David Leszkowitz

**Affiliations:** 1 Family Medicine, Larkin Community Hospital Palm Springs Campus, Hialeah, USA; 2 Family Medicine, Medficient Health Systems, Laurel, USA; 3 Family Medicine, Lakeside Medical Center, Belle Glade, USA; 4 Dermatology, University of Medical Sciences (UNIMED), Ondo State, NGA; 5 Medicine and Surgery, University of Nigeria, Nsukka, Enugu, NGA; 6 Internal Medicine, University of Ghana School of Medicine and Dentistry, Accra, GHA; 7 Department of Medicine, All Saints University School of Medicine, Roseau, DMA; 8 Family Medicine, Nnamdi Azikiwe University, Nnewi, NGA; 9 Emergency Department, Bader Al Janoub General Hospital, Najran, SAU; 10 Internal Medicine, International American University College of Medicine, Vieux Fort, LCA; 11 Family Medicine, Federal Medical Centre, Jabi Abuja, Abuja, NGA; 12 Substance Use and Addiction, Larkin Community Hospital Palm Springs Campus, Hialeah, USA

**Keywords:** cdc-wonder database, mental and behavioral disorders, psychoactive substance, mortality rate, epidemiological trends

## Abstract

Background

The persisting challenge of substance use disorder’s impact on society prompts the need for insights into its mortality trends. This study examines epidemiological patterns and factors tied to mortality rates in individuals with psychoactive substance-related mental and behavioral disorders from 1999 to 2020.

Methodology

This study used a retrospective observational design. The study analyzed the Centers for Disease Control and Prevention’s (CDC) Wide-ranging Online Data for Epidemiologic Research (WONDER) database information, encompassing mortality and population-based data. Data extraction focused on specific criteria such as age, sex, race/ethnicity, and geographic location. Descriptive statistics were employed to depict the study population and reveal epidemiological trends.

Results

The study encompassed data from 239,573 individuals who died due to psychoactive substance use-related mental and behavioral disorders between 1999 and 2020. The overall mortality rate was 3.55 per 100,000 individuals (95% confidence interval (CI) = 3.55-3.54) across the study period. Noticeable discrepancies in mortality rates emerged among various geographic regions, genders, races, and age groups. Males (5.32 per 100,000 individuals) experienced notably higher mortality rates than females (1.80 per 100,000 individuals), while the 55-64 age group faced elevated mortality risks (8.24 per 100,000 individuals) compared to the 25-34 age group (1.71 per 100,000 individuals). Significant variations in mortality rates were also evident across different racial and ethnic groups.

Conclusions

Between 1999 and 2020, the study explored mortality rates in individuals with psychoactive substance use-related mental and behavioral disorders. This analysis revealed variations in mortality across genders, ages (with the 55-64 age group facing higher risks compared to the 25-34 age group), and ethnicities. Notably, males exhibited higher mortality than females. Additionally, divergent rates were observed among different geographic regions. These insights can inform public health initiatives and support the development of targeted strategies to reduce mortality rates and improve the well-being of affected individuals.

## Introduction

Psychoactive substance use has long been a global public health concern, with profound implications for mental and behavioral disorders. The abuse and misuse of substances such as alcohol, opioids, stimulants, and other illicit drugs have been linked to adverse outcomes, including severe mental health disorders and increased mortality rates [1,2]. Understanding the epidemiological trends and factors associated with mortality in individuals with psychoactive substance use-related mental and behavioral disorders is crucial for devising effective prevention strategies and targeted interventions [3]. Based on the National Center for Drug Abuse Statistics (NCDAS), as of 2020, there were approximately 37.309 million individuals aged 12 years and older in the United States (US) who were current illegal drug users. This accounts for around 13.5% of Americans aged 12 and over, indicating a 3.8% rise compared to the previous year. The data also highlights a concerning pattern in synthetic opioid (excluding methadone) deaths, which experienced a dramatic 264% surge between 2012 and 2015 in the US [4].

Psychoactive substances directly affect physiological systems, leading to acute and chronic health complications. For instance, opioids, alcohol, and stimulants can depress respiratory function, potentially causing respiratory failure or overdose. Chronic use of these substances can harm vital organs such as the liver, heart, and brain, increasing the risk of organ failure and mortality [5,6]. Additionally, psychoactive substances can alter brain function and neurotransmitter systems, resulting in changes in mood, behavior, and cognition. Prolonged use may lead to neuroadaptive changes, contributing to addiction and mental health disorders such as depression, anxiety, and psychosis. These mental health issues can heighten the risk of self-harm, suicide, and accidental deaths [6,7].

The devastating consequences of psychoactive substance use are well-documented, affecting not only the individuals involved but also their families, communities, and society as a whole. The rise in substance-related disorders and the associated mortality rates have become pressing challenges for healthcare systems worldwide [6,8]. This study aims to investigate the mortality rate in individuals with mental and behavioral disorders associated with psychoactive substance use through a comprehensive analysis of the Centers for Disease Control and Prevention’s (CDC) Wide-ranging Online Data for Epidemiologic Research (WONDER) database. The CDC-WONDER database offers a rich repository of nationwide data, providing researchers with valuable insights into the patterns of substance use and its consequences on public health [9].

This study aimed to investigate and analyze the temporal patterns and changes in the mortality rate of psychoactive substance use-related mental and behavioral disorders over a specified period. It also aimed to assess the hypothesis that specific demographic factors, such as age, gender, and socioeconomic status, are associated with variations in mortality rates among individuals with psychoactive substance use-related disorders.

## Materials and methods

Study design and sample

In this study, a retrospective observational design was employed, and data from the CDC-WONDER database was analyzed to examine epidemiological trends and factors associated with mortality rates in individuals with psychoactive substance use-related mental and behavioral disorders. The study population consisted of individuals who had been diagnosed with mental and behavioral disorders related to psychoactive substance use and had experienced mortality due to these conditions. The database primarily focused on mortality data obtained from death certificates, providing information on the place of death and patient-specific demographics, including details about the cause of death, demographic factors, and other relevant information.

Data source

The primary data source for this study was the CDC-WONDER database, a publicly accessible online resource maintained by the CDC. This database contained a wide range of mortality data, including information on the cause of death, age, sex, geographic location, and other demographic variables, covering a diverse and representative sample of the US population. The study examined all-cause mortality and cause-specific mortality rates for mental and behavioral disorders due to the use of psychoactive substances, using specific International Classification of Disease (ICD-10) codes: F10 (use of alcohol), F11 (use of opioids), F12 (use of cannabinoids), F13 (use of sedatives or hypnotics), F14 (use of cocaine), F15 (use of other stimulants, including caffeine), F16 (use of hallucinogens), F17 (use of tobacco), F18 (use of volatile solvents), and F19 (use of other psychoactive substances). Cases with incomplete data regarding psychoactive substance use, mental and behavioral disorders, mortality rates, or key covariates were excluded. The mean or median imputation method was used to address the missing data.

Data extraction and analysis

Data extraction from the CDC-WONDER database was performed using predefined criteria and variables. The information collected included demographic characteristics such as age, sex, race/ethnicity, and geographic location. Additionally, specific details on the types of psychoactive substances involved, comorbidities, and the underlying cause of death were extracted. Descriptive statistics were employed to characterize the study population and identify epidemiological trends in psychoactive substance use-related mental and behavioral disorders leading to mortality. Aggregate data for the selected time period (1999-2020) and available patient characteristics were summarized, presenting mortality rates per 100,000 people and the total number of deaths over the years for all patients. Sample mortality rates associated with 95% confidence intervals (CIs) were computed, assuming normality. No adjustments were made for multiple comparisons or unavailable data and only available patients’ data were utilized in all tables and figures. All statistical analyses were conducted using Microsoft Excel version 2019 (Microsoft Corp., Redmond, WA, USA).

Ethical considerations

In this study, as all the data used were publicly available and contained no personally identifiable information, obtaining informed consent and ethics committee approval was not required.

## Results

Aggregate data for 239,573 individuals who died of mental and behavioral disorders associated with psychoactive substance use between 1999 and 2020 were obtained. The overall mortality rate per 100,000 people during this period was reported as 3.55 (95% CI = 3.55-3.54). The highest mortality rates were recorded in 2020, with a rate of 5.93 (95% CI = 5.93-5.85), followed by 2019 with a rate of 4.62 (95% CI = 4.62-4.55), and 2018 with a rate of 4.40 (95% CI = 4.4-4.33) (Table 1).

**Table 1:** Year-wise mortality rate trends for demographic characteristics (1999-2020).

		Mortality rate per 100,000																						
	Variables	1999	2000	2001	2002	2003	2004	2005	2006	2007	2008	2009	2010	2011	2012	2013	2014	2015	2016	2017	2018	2019	2020	Average (1999-2020)
Overall data	Total number of deaths	8,619	8,721	9,087	9,528	10,104	10,683	11,271	11,816	8,416	8,147	8,130	8,540	8,898	9,371	10,048	10,569	11,617	13,072	13,839	14,390	15,163	19,544	-
	Total Population	279M	281M	285M	288M	290M	293M	296M	298M	301M	304M	307M	309M	312M	314M	316M	319M	321M	323M	326M	327M	328M	329M	-
	Overall mortality rate	3.09	3.1	3.19	3.31	3.48	3.65	3.81	3.96	2.79	2.68	2.65	2.77	2.86	2.99	3.18	3.31	3.61	4.05	4.25	4.4	4.62	5.93	3.53
	95% Confidence interval	3.09 - 3.02	3.1 - 3.03	3.19 - 3.12	3.31 - 3.25	3.48 - 3.41	3.65 - 3.58	3.81 - 3.74	3.96 - 3.89	2.79 - 2.73	2.68 - 2.62	2.65 - 2.59	2.77 - 2.71	2.86 - 2.8	2.99 - 2.92	3.18 - 3.12	3.31 - 3.25	3.61 - 3.55	4.05 - 3.98	4.25 - 4.18	4.4 - 4.33	4.62 - 4.55	5.93 - 5.85	-
	Standard error	0.03	0.03	0.03	0.03	0.03	0.04	0.04	0.04	0.03	0.03	0.03	0.03	0.03	0.03	0.03	0.03	0.03	0.04	0.04	0.04	0.04	0.04	-
Gender	Male	4.83	4.80	4.89	5.09	5.32	5.59	5.74	5.91	4.25	4.08	4.00	4.25	4.31	4.47	4.76	4.94	5.34	6.07	6.35	6.45	6.79	8.90	5.32
	Female	1.42	1.46	1.55	1.60	1.71	1.77	1.95	2.07	1.38	1.32	1.34	1.33	1.45	1.54	1.64	1.74	1.94	2.09	2.21	2.41	2.51	3.06	1.80
Race	White	2.91	2.97	3.05	3.24	3.46	3.66	3.89	4.01	2.85	2.78	2.73	2.92	3.03	3.2	3.42	3.54	3.92	4.4	4.62	4.75	4.99	6.4	3.67
	Black or African American	4.73	4.55	4.64	4.45	4.27	4.29	4.21	4.45	3.16	2.77	2.86	2.71	2.66	2.56	2.73	2.88	2.98	3.32	3.51	3.67	3.92	5.13	3.66
	Asian or Pacific Islander	0.45	0.39	0.41	0.64	0.56	0.69	0.72	0.91	0.48	0.37	0.45	0.37	0.45	0.51	0.55	0.66	0.60	0.62	0.87	0.83	0.89	1.24	0.62
	American Indian or Alaska Native	7.38	6.10	7.26	6.15	7.91	7.70	6.86	7.59	4.91	5.05	4.68	4.20	4.60	5.37	4.85	6.40	6.36	6.67	6.85	8.65	8.92	10.88	6.61
Age	25-34 years	1.6	1.6	1.6	1.5	1.6	1.7	1.6	1.8	1.2	1.0	0.9	1.2	1.1	1.3	1.4	1.6	1.9	2.3	2.3	2.5	2.5	3.4	1.71
	35-44 years	4.72	4.55	4.49	4.91	4.87	4.62	4.70	4.71	3.05	2.87	2.57	2.56	2.70	2.63	2.88	3.09	3.27	3.76	4.01	4.35	4.99	6.60	3.95
	45-54 years	6.58	6.75	7.21	7.44	7.93	8.26	8.60	8.72	6.38	6.08	5.98	6.13	6.42	6.21	6.36	6.61	7.09	7.43	7.99	7.91	8.04	10.40	7.30
	55-64 years	6.37	6.25	6.55	6.80	6.98	7.43	7.94	8.04	6.52	6.44	6.33	6.66	6.82	7.58	8.21	8.40	8.96	10.31	10.73	11.22	11.65	15.04	8.24
	65-74 years	5.40	5.46	5.40	5.67	5.92	6.36	6.01	6.48	4.61	4.42	4.85	4.84	4.81	5.10	5.55	5.49	6.35	7.02	7.47	7.55	8.12	10.10	6.04
	75-84 years	4.91	4.66	4.58	4.34	4.68	5.37	5.58	6.28	3.74	3.34	3.46	3.58	3.42	3.90	4.16	4.04	3.95	4.25	4.33	4.88	4.89	5.46	4.45
	85+ years	3.80	4.10	4.06	3.87	4.55	4.95	7.33	7.30	3.00	3.12	2.70	3.35	3.10	2.92	2.88	3.39	3.69	3.97	3.59	3.80	3.48	3.98	3.95
Hispanic origin	Hispanic or Latino	2.43	2.27	2.16	2.35	2.37	2.21	2.53	2.45	1.59	1.41	1.38	1.42	1.31	1.37	1.54	1.62	1.64	1.80	1.98	2.05	2.26	3.07	1.96
	Not Hispanic or Latino	3.13	3.18	3.30	3.42	3.63	3.86	4.01	4.20	2.99	2.90	2.87	3.00	3.14	3.28	3.48	3.63	3.98	4.49	4.71	4.89	5.12	6.55	3.81

Mortality rate of psychoactive substance use-related mental disorders based on location

**Figure 1:**
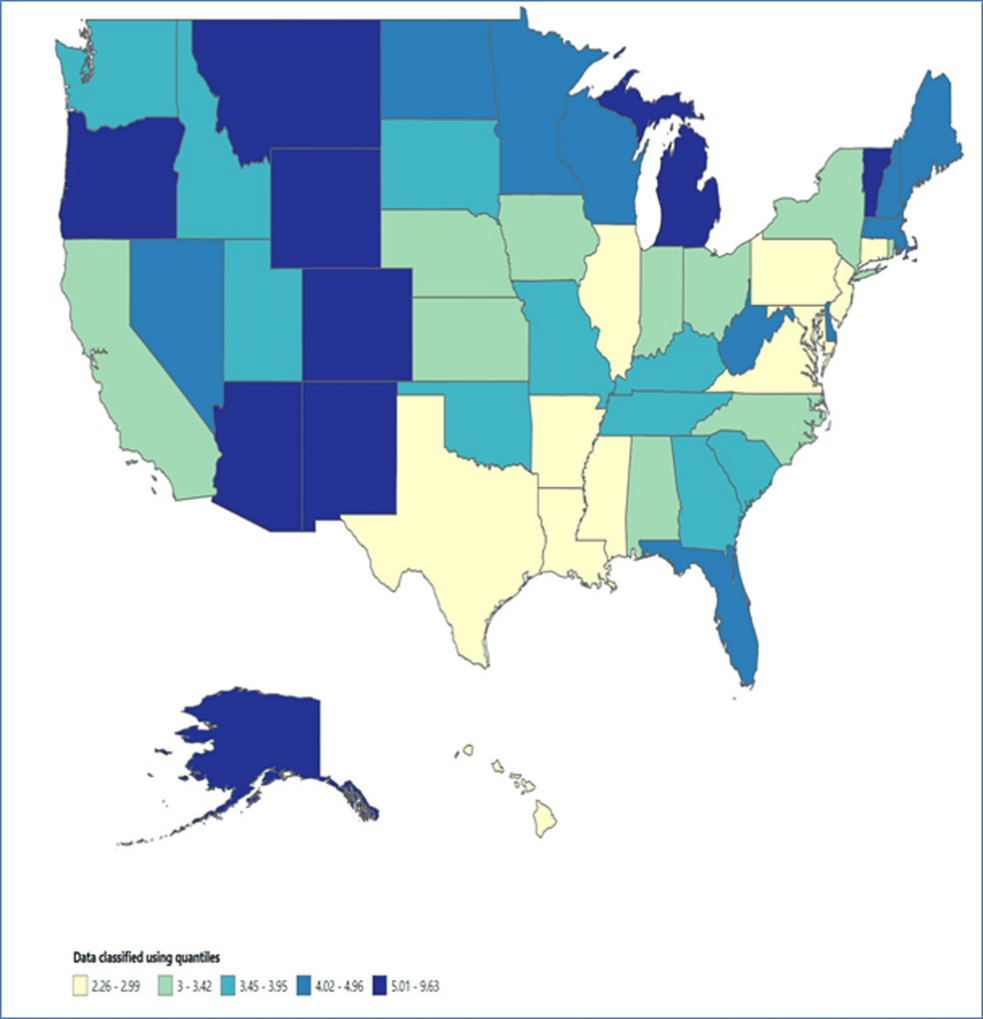
Mortality rate across the US states (1999-2020).

The analysis revealed significant regional disparities in mortality rates among individuals with psychoactive substance use-related mental and behavioral disorders. States such as New Mexico (9.63 per 100,000; 95% CI = 9.34-9.92), Alaska (8.99 per 100,000; 95% CI = 8.52-9.47), Oregon (8.25 per 100,000; 95% CI = 8.06-8.45), District of Columbia (8.21 per 100,000; 95% CI = 7.73-8.69), and Montana (6.71 per 100,000; 95% CI = 6.37-7.06) exhibited considerably higher mortality rates compared to other states. Table 2 below displays the data depicting the trend in mortality rates categorized by location, encompassing four census regions, nine census divisions, ten health and human services (HHS) regions, and the mortality rates in the leading five states. The geographic variations observed in mortality rates underscore the importance of considering location-specific factors and tailoring interventions to address the unique challenges faced by different communities (Figure 1).

**Table 2:** Mortality rate trend based on census region, census division, HHS region, and states (1999-2020). *: top five selected states and counties with the highest mortality rates are presented in the table. HHS: Health and Human Services; AL: Alabama; AK: Alaska, AZ: Arizona; AR: Arkansas; CA: California; CO: Colorado; CT: Connecticut; DE: Delaware; FL: Florida; GA: Georgia; HI: Hawaii, ID: Idaho; IL: Illinois; IN: Indiana; IA: Iowa; KS: Kansas; KY: Kentucky; LA: Louisiana; ME: Maine; MD: Maryland; MA: Massachusetts; MI: Michigan; MN: Minnesota; MS: Mississippi; MO: Missouri; MT: Montana; NE: Nebraska; NV: Nevada; NH: New Hampshire; NJ: New Jersey; NM: New Mexico; NY: New York; NC: North Carolina; ND: North Dakota; OH: Ohio; OK: Oklahoma; OR: Oregon; PA: Pennsylvania; RI: Rhode Island; SC: South Carolina; SD: South Dakota; TN: Tennessee; TX: Texas; UT: Utah; VT: Vermont; VA; Virginia; WA: Washington; WV: West Virginia; WI: Wisconsin; WY: Wyoming

Variables		Number of deaths	Mortality rate per 100,000	95% confidence interval
Census region	Census region 1: Northeast	39964	3.29	3.26-3.33
	Census region 2: Midwest	52312	3.57	3.54-3.6
	Census region 3: South	80157	3.21	3.19-3.23
	Census region 4: West	67140	4.28	4.25-4.31
Census division	Division 1: New England	13221	4.17	4.17-4.09
	Division 2: Middle Atlantic	26743	2.99	2.99-2.95
	Division 3: East North Central	36324	3.57	3.57-3.53
	Division 4: West North Central	15988	3.56	3.56-3.51
	Division 5: South Atlantic	45928	3.52	3.52-3.49
	Division 6: East South Central	14291	3.57	3.57-3.51
	Division 7: West South Central	19938	2.51	2.51-2.48
	Division 8: Mountain	25850	5.41	5.41-5.35
	Division 9: Pacific	41290	3.78	3.78-3.74
HHS region	HHS region #1: CT, ME, MA, NH, RI, VT	13221	4.17	4.09-4.24
	HHS region #2: NJ, NY	19862	3.21	3.17-3.26
	HHS region #3: DE, DC, MD, PA, VA, WV	18451	2.83	2.79-2.88
	HHS region #4: AL, FL, GA, KY, MS, NC, SC, TN	48649	3.66	3.62-3.69
	HHS region #5: IL, IN, MI, MN, OH, WI	41017	3.62	3.58-3.65
	HHS region #6: AR, LA, NM, OK, TX	24174	2.88	2.85-2.92
	HHS region #7: IA, KS, MO, NE	9899	3.31	3.24-3.37
	HHS region #8: CO, MT, ND, SD, UT, WY	12021	5.07	4.98-5.16
	HHS region #9: AZ, CA, HI, NV	37060	3.56	3.52-3.6
	HHS region #10: AK, ID, OR, WA	15219	5.42	5.34-5.51
State*	Alaska	1376	8.99	8.52-9.47
	District of Columbia	1120	8.21	7.73-8.69
	Montana	1454	6.71	6.37-7.06
	New Mexico	4236	9.63	9.34-9.92
	Oregon	6927	8.25	8.06-8.45

Mortality rate of psychoactive substance use-related mental disorders based on gender

**Figure 2:**
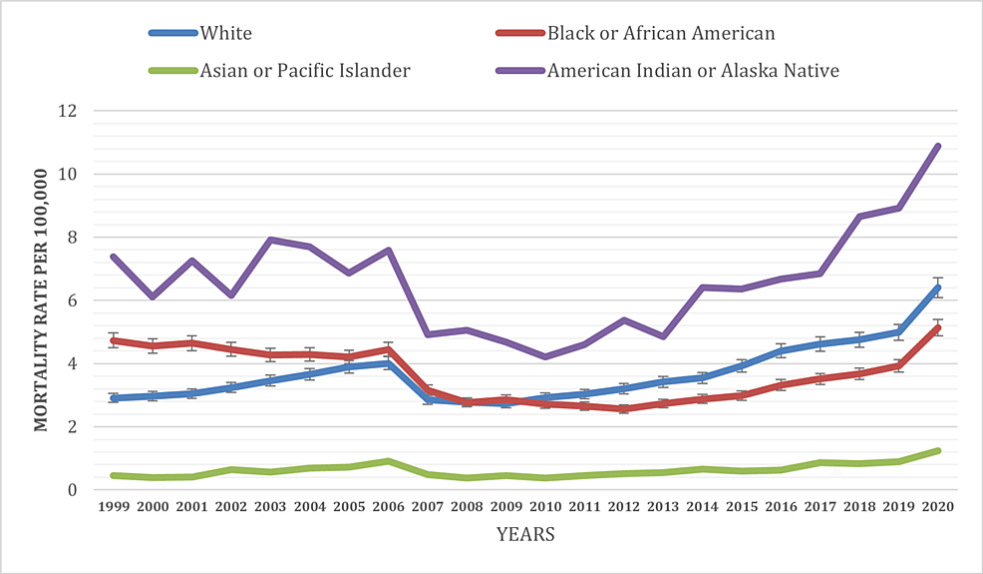
Mortality rate based on gender classification (1999-2021).

Gender was identified as a significant factor associated with mortality rates in individuals with psychoactive substance use-related mental and behavioral disorders. Males exhibited a significantly higher mortality rate of 5.32 per 100,000 people compared to 1.80 per 100,000 people for females during the study period. Additionally, an increasing trend in the mortality rate was observed in both genders over the time period from 1991 to 2021. In 1991, the mortality rate for males was 4.83 per 100,000 people, while for females, it was 1.42 per 100,000 people. By 2021, the mortality rate had risen to 8.90 per 100,000 people for males and 3.06 per 100,000 people for females (Figure 2). The disparity in mortality risk highlights the necessity of gender-sensitive approaches in prevention, early intervention, and treatment strategies targeting substance use disorders.

Mortality rate of psychoactive substance use-related mental disorders based on race and ethnicity

**Figure 3:**
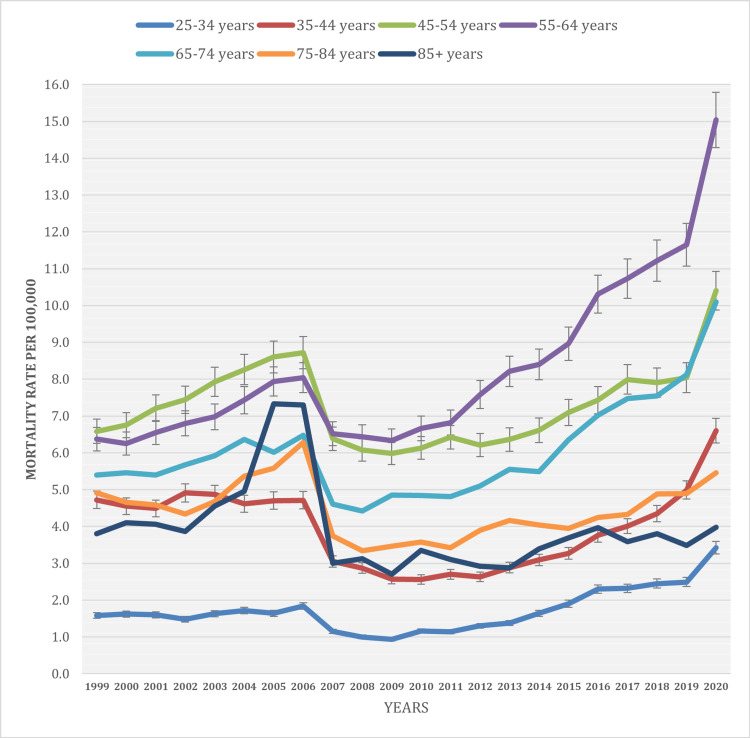
Mortality rate based on type of race (1999-2021).

The analysis revealed notable differences in mortality rates based on race and ethnicity among individuals with psychoactive substance use-related mental and behavioral disorders. During the reported data from 1999 to 2020, American Indian/Alaska Native adolescents had the highest mortality rate at 6.61 per 100,000 people, followed by White individuals at 3.67 per 100,000 people, Black or African American individuals at 3.67 per 100,000 people, Non-Hispanic individuals at 3.81 per 100,000 people, and Hispanic individuals at 1.96 per 100,000 people. Asian or Pacific Islander individuals had the lowest mortality rate at 0.62 per 100,000 people (Table 1). Furthermore, significant variations in mortality rates were observed based on racial and ethnic composition over the time period of 1991 to 2021 (Figure 3).

Mortality rate of psychoactive substance use-related mental disorders based on age

**Figure 4:**
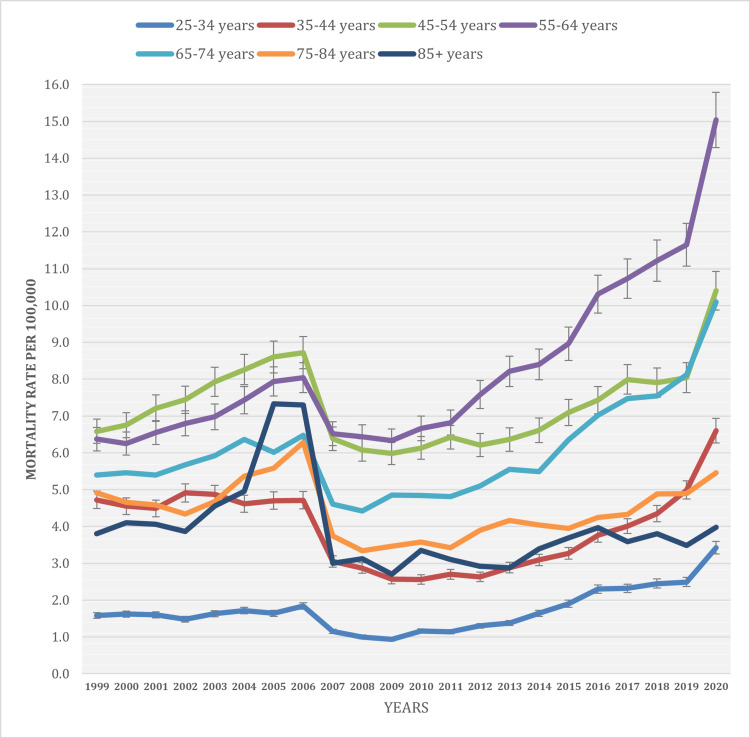
Mortality rate age classification (1999-2021).

Age was a significant factor associated with mortality rates in individuals with psychoactive substance use-related mental and behavioral disorders. The study found that mortality rates associated with mental and behavioral disorders linked to psychoactive substance use varied significantly across different age groups (Figure 4). During the reported data from 1999 to 2020, ages between 55 and 64 years had the highest mortality rate at 8.24 per 100,000 people, followed by the 45-54 years age group (7.30 per 100,000 people), the 65-74 years age group (6.04 per 100,000 people), the 75-84 years age group (4.45 per 100,000 people), the 35-44 years age group (3.95 per 100,000 people), and the 85+ years age group (3.95 per 100,000 people) (Table 1). The 25-34 years age group recorded a mortality rate of 1.71 per 100,000 people, which was the lowest among all age groups.

## Discussion

The present study aimed to explore epidemiological trends and factors associated with mortality rates in individuals with psychoactive substance use-related mental and behavioral disorders using data from the CDC-WONDER database. Our analysis revealed several key findings based on age, gender, age group, and race, shedding light on the complex interplay of these factors in relation to mortality rates. The findings provide valuable insights into the complex interactions between these variables and their impact on mortality rates.

Location emerged as a critical factor influencing mortality rates in individuals with psychoactive substance use-related disorders. Significant regional disparities were observed, with certain states exhibiting considerably higher mortality rates compared to others. States such as New Mexico, Alaska, Oregon, District of Columbia, and Montana reported the highest mortality rates. Based on the data published by the NCDAS, West Virginia has reported the highest rate of overdose deaths, with 51.5 deaths per 100,000 people. Delaware closely follows with 43.8 deaths per 100,000 people, and Maryland reports 37.2 deaths per 100,000 people [4]. Similar findings were observed in another retrospective study conducted by Glei et al. Utilizing the National Center for Health Statistics database, it was found that the highest drug-associated mortality rate among US states was observed in West Virginia [10]. However, these findings do not align with the current study’s results, as this study was not specifically conducted to examine substance use-related mental disorders. Therefore, further analysis is needed to understand the variations in mortality rates related to psychoactive substance use and mental disorders based on location. These variations underscore the importance of considering location-specific factors and tailoring interventions to address the unique challenges faced by different communities. Targeted efforts to improve access to mental health services, substance use disorder treatment, and prevention programs in regions with elevated mortality rates can play a pivotal role in reducing the burden of substance use-related mortality.

Gender was also identified as a significant determinant of mortality rates in this population. Males exhibited significantly higher mortality rates compared to females. This gender-specific variation suggests that males may face greater mortality risk when experiencing mental and behavioral disorders associated with psychoactive substances compared to females. These findings align with a prior analysis conducted by Svensson et al., which revealed that male patients with substance use disorder faced a mortality risk that was 1.41 to 1.59 times higher than their female counterparts [10-13]. The gender differences in mortality rates highlight the need for gender-sensitive approaches in prevention, early intervention, and treatment strategies targeting substance use disorders. Understanding the underlying factors contributing to this disparity can inform the development of targeted interventions to improve outcomes for both male and female individuals.

Race and ethnicity demonstrated notable associations with mortality rates among individuals with psychoactive substance use-related disorders. American Indian/Alaska Native adolescents had the highest mortality rate, followed by White, Black or African American, Non-Hispanic, and Hispanic individuals. Asian or Pacific Islander individuals had the lowest mortality rate. These results are consistent with the Morbidity and Mortality Weekly Report’s discovery, which showed that in 2015, American Indians/Alaska Natives experienced the highest drug overdose death rates [14]. However, some contrasting findings were also reported in earlier studies. Consequently, further analysis is necessary to comprehend the disparities in mortality rates concerning psychoactive substance use and mental disorders based on race [15,16]. These findings emphasize the significance of considering cultural factors and tailoring suicide prevention efforts to address the unique challenges faced by various racial and ethnic groups. Culturally sensitive approaches that take into account the specific needs and experiences of different racial and ethnic communities can contribute to more effective interventions and ultimately reduce mortality rates.

The analysis of mortality rates based on age groups revealed intriguing patterns. Individuals aged 55-64 years exhibited the highest mortality rate, followed by the 45-54 years age group, the 65-74 years age group, the 75-84 years age group, and individuals aged 85 and above. The 35-44 years age group displayed the lowest mortality rate. Nevertheless, these findings align with earlier studies, and additional research is warranted to comprehend the mortality rate patterns among different age groups [10,14,17]. These findings highlight the varying impact of psychoactive substance use-related disorders across different age groups. Tailored interventions for each age cohort, considering their unique risk factors and needs, are essential to effectively address the mental health challenges associated with substance use disorders. This study offers crucial insights into the factors influencing mortality rates among individuals with psychoactive substance use-related mental and behavioral disorders, accounting for location, gender, race, and age group. The results emphasize the need for nuanced, targeted interventions addressing the complexities of substance use disorders and diverse individual needs. Tailoring strategies to specific risk factors associated with each demographic variable can aid healthcare professionals and policymakers in reducing mortality rates and enhancing the mental well-being of those affected. Nevertheless, the study’s limitations warrant acknowledgment, including potential confounding variables and generalizability to other populations. Collaborative, interdisciplinary research remains imperative for a comprehensive understanding of this public health concern and the development of evidence-based preventive and interventional strategies.

Despite its significance, this study’s limitations should be recognized. One notable limitation of this study is that the analysis primarily focused on the relationship between psychoactive substance use and mortality rates without accounting for the potential influence of these confounding factors. The data used, derived from CDC-WONDER death certificates, may suffer from underreporting or misclassification of causes of death. Additionally, the study’s focus on the US population restricts generalizability to regions with different healthcare systems and cultural norms. Lack of detailed substance use and comorbidity information may hinder a comprehensive grasp of mortality factors. The retrospective observational design further prevents establishing causal relationships. Nevertheless, the study proves invaluable in highlighting epidemiological trends and factors impacting mortality rates in psychoactive substance use-related disorders, guiding future research and public health initiatives. The study benefits from the CDC-WONDER database’s comprehensive mortality and population data, encompassing a diverse sample of the US population. The extensive 1999-2020 timeframe provides valuable insights into long-term trends. Predefined criteria and variables ensure data extraction accuracy. The findings on location, gender, race, and age group inform targeted interventions to mitigate mortality rates and enhance mental well-being for vulnerable groups.

## Conclusions

Between 1999 and 2020, the study explored mortality rates in individuals with psychoactive substance use-related mental and behavioral disorders. This analysis revealed variations in mortality across genders, ages (with the 55-64 age group facing higher risks compared to the 25-34 age group), and ethnicities. Notably, males exhibited higher mortality than females. Additionally, divergent rates were observed among different geographic regions. These insights can inform public health initiatives and support the development of targeted strategies to reduce mortality rates and improve the well-being of affected individuals. This study, while significant, acknowledges limitations. CDC-WONDER death certificate data may face underreporting or misclassification. The US-centric focus limits generalizability to diverse healthcare systems and cultures. Nevertheless, the study, leveraging the CDC-WONDER database, offers valuable insights into long-term trends from 1999 to 2020. Defined criteria and variables ensure accuracy, informing targeted interventions for vulnerable groups and guiding future research and public health initiatives.
